# Molecular docking analysis of thiazo inhibitors with the virulent factor cystalysin from *Treponema denticola*

**DOI:** 10.6026/97320630019094

**Published:** 2023-01-01

**Authors:** Serafina Andrew, Kavitha Sankaran, Surya Sekaran, Gayathri Rengasamy, Vishnu Priya Veeraraghavan, Rajalakshmanan Eswaramoorthy

**Affiliations:** 1 Department of Biochemistry, Saveetha Dental College and Hospitals, Saveetha Institute of Medical and Technical Sciences, Saveetha University, Chennai-600077; 2 Department of Biomaterials (Green lab), Saveetha Dental College and Hospital, Saveetha Institute of Medical and Technical Science (SIMATS), Saveetha University, Chennai-600077

**Keywords:** Antimicrobial agents, cystalysin, *Treponema denticola*, Periodontitis, ADMET, molecular docking

## Abstract

*Treponema Denticola* has a virulent protein called cystalysin, which causes periodontitis. Therefore, it is of interest to design efficient drug that may have fewer side effects than the present clinical drugs, considering most of them are multidrug resistant. The molecular docking analysis show that the selected thiazo derivatives (1-6) show better binding energies and amino acid interactions compared to the clinically proven drugs proving to be potential inhibitors against the protein.

## Background:

*Treponema denticola* is an oral anaerobic spirochete which helps in the progression of periodontitis [3]. Periodontitis is a gum disease which can destroy the surrounding tissue around your teeth. If the disease is left untreated it can spread to and ruin the alveolar bone, eventually leading to loss of teeth, causing irreparable damage. Therefore, it is of relevance to find a more efficient drug that may have fewer side effects than the present clinical drugs used today to treat periodontitis today. Since there is a significant link between periodontal bacteria and other illnesses like diabetes, rheumatoid arthritis, or cardiovascular disease, it is evident that an effective periodontal cure would be beneficial to overall health [1]. It is an undefined series of microbial infections with more than 300 species of bacteria that are now known to inhabit the oral cavity as its primary cause. *Treponema denticola* is one of the bacteria involved in the development of periodontitis [2]. The high presence of *Treponema denticola* and other proteolytic Gram-negative bacteria in periodontal pockets may be a significant factor in the development of periodontal disease. The buildup of these bacteria and their by-products in the pocket could make the periodontal surface lining cells extremely vulnerable to lysis and injury. According to research, T. denticola can cling to fibroblasts, epithelial cells, and extracellular matrix elements found in periodontal tissues. It can also release a number of harmful substances that could increase the bacteria's pathogenicity [4]. Examining the *Treponema denticola* genome shows mechanisms that mediate coaggregation, cell signaling, stress protection, and other competing and cooperative action which are consistent with the pathogenic behavior and environment of subgingival dental plaque [5]. Cystalysin is a lyase that is found in *Treponema denticola* and its function is to catabolize L-cysteine to create pyruvate, ammonia, and H2S, which allows the bacterium to produce sulfide, which is in charge of hemolytic and hemoxidative processes, as well as the destruction of gingival and periodontal tissue. Cystalysin belongs to a new class of virulence factors that are dependent on pyridoxal 5'-phosphate (PLP) and can cause cell lysis [6]. Therefore, it is of interest to document the potential thiazo antimicrobial compound targeting the virulence factor cystalysin in *Treponema denticola*.

## Material and Methods:

## Protein preparation:

**Figure 1:**
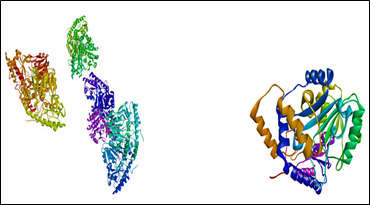
3D structure of gingipain R protein (PDB ID: 1CVR)

The 3D crystal structure of the cystalysin protein (PDB ID: 1C7N) was downloaded from the protein data bank (Figure 1). As per standard protocol, protein preparation was done using the software Biovia Discovery Studio and Mgl tools 1.5.7. Water molecules and cofactors were chosen for elimination. The previously connected ligands were removed, and the protein was produced by adding polar hydrogens and Kollmans charges with Auto Prep.

## Ligand preparations:

**Figure 2:**
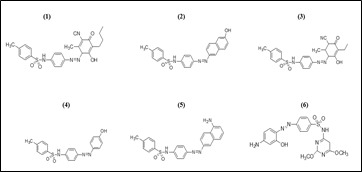
2D Structures of the imidazole quinolines compounds (1-6)

The 2D structures of the literature derived thiazo compounds are drawn using the ChemDraw 16.0 software (Figure 2). During the optimization method, the software Chem3D was employed and all parameters were selected in order to achieve a stable structure with the least amount of energy. The structural optimization approach was used to estimate the global lowest energy of the title chemical. Each molecule's 3D coordinates (PDB) were determined using optimized structure.

## Auto dock Vina analysis:

**Figure 3:**
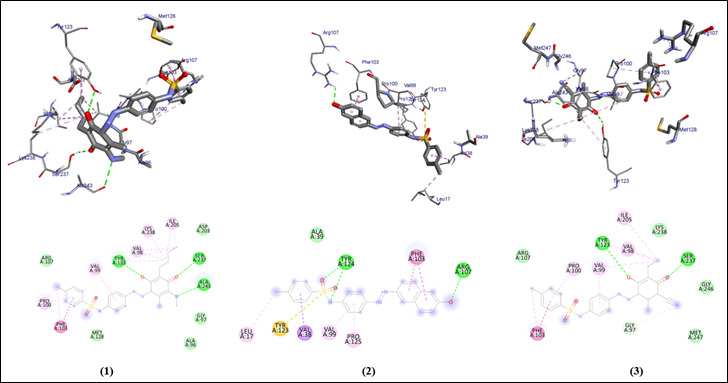
Molecular docking analysis of compounds (1-3) against the target gingipain R of *Porphyromonas gingivalis*

**Figure 4:**
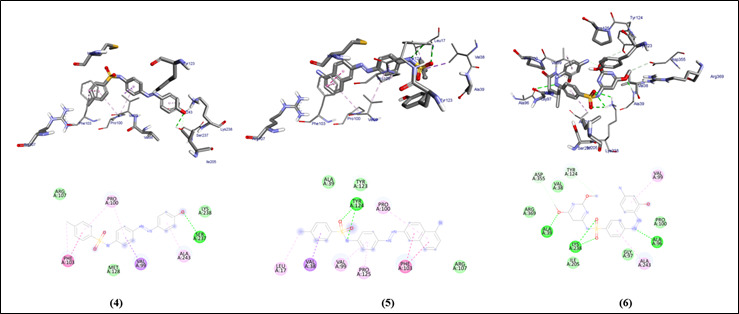
Molecular docking analysis of compounds (4-6) against the target gingipain R of *Porphyromonas gingivalis*

The graphical user interface Auto Dock vina was used for Ligand-Protein docking interactions (Figure 3, Figure 4). Auto Dock Tools (ADT), a free visual user interface (GUI) for the AutoDock Vina software, was used for the molecular docking research. The grid box was built with dimensions 27.1237, 18.5722, 40.9884 pointing in the x, y, and z axes. The central grid box for 1C7N was 11.0615, 0.3017,49.9026 A. For each ligand, nine alternative conformations were created and ranked based on their binding energies utilizing Auto Dock Vina algorithms.

## In-Silico drug likeness and toxicity predictions:

In the present study, in-silico pharmacokinetic properties (ADME), drug-likeness, toxicity profiles are examined using SwissADME, and ProTox-II online servers. The ADME parameters involve the absorption, distribution, metabolism and estimation of a drug [7]. The SwissADME, a web tool from Swiss Institute of Bioinformatics (SIB) is used to convert the two-dimensional structures into their simplified molecular input line entry system (SMILES). The physicochemical properties (molar refractivity, topological polar surface area, number of hydrogen bond donors/ acceptors); pharmacokinetics properties (GI absorption, BBB permeation, P-gp substrate, cytochrome-P enzyme inhibition, skin permeation (log Kp)) which are critical parameters for prediction of the absorption and distribution of drugs within the body, and drug likeness (Lipinski's rule of five) were predicted using SwissADME. The toxicological endpoints (Hepatotoxicity, Carcinogenicity, Immunotoxicity, Mutagenicity) and the level of toxicity (LD50, mg/Kg) are determined using the ProTox-II server.

## Statistical analysis:

One way ANOVA was used for statistical analysis. The clinically proven drugs are used as a control and the results are compared. The significance of the results was found to be p < 0.05

## Results:

## Molecular docking interaction of thiazo compounds against of cystalysin protein of *Treponema denticola*:

All the compounds (1-6) are run against the target of cystalysin protein of *Treponema denticola* and it shows the range between -6.6 to -8.4 (Table 1). The compounds show hydrogen molecules interaction similar to clinically proven drugs (azithromycin, sulfanilamide and sulfamethoxazole). Clinically proven drugs show amino acid interaction within the binding site of protein similar to the selected compounds (1-6). All the compounds show better binding affinity within the binding site compared to the control drugs.

## SwissADME and Lipinski's rule of five:

The compounds show log Kp values between -4.96 to -8.04 cm/s. It should be noted that more the negative value, more the skin permeation (Table 2). All the compounds (1-6) show low gastro intestinal absorption so it needs a carrier molecule. Compounds (1-6) show no blood brain barrier permeability. All the compounds (1-5) except compound 6 obey Lipinski's rule of five similar to control groups (Table 3).

## Toxicity profiling:

The compounds show class 4, 5, and 6 in toxicity. The compounds 2, 4 and 5 show a similar LD50 value (5000mg/kg). Compounds 4, 5, and 6 are inactive in hepatotoxicity, immunotoxicity, mutagenicity and cytotoxicity. None of the compounds are cytotoxic (Table 4).

## Discussion:

Compounds (1-5) show better interaction within the protein binding site with least binding score (-7.4 to -8.4 kcal/mol) as shown in Table 1. Compared to clinical drugs, Azithromycin and Sulfamethoxazole (-4.7 to -6.9 kcal/mol), all the selected compounds showed better docking scores. The ADMET profile shows all compounds are obeying Lipinski's rule of five except compound 6. Their profiles are similar to clinically proven drugs [8,1^9^, ^10^,^11^,^12^,^13^,^14^,^15^, ^16^]. All the selected compounds show toxicity and profile similar to Sulfamethoxazole and better LD50 value (<1000 mg/kg). The ligands show low Gl absorption and no BBB permeation. The databases used such as Protein Data Bank, Auto dock vina and Swiss ADME are good for prototype development and small academic research and experiments but they are far away from the requirements of drug analysis and discovery in real life situations. However, docking strategies have undoubtedly become more sophisticated, they still suffer from high false-positive rates. Computer assisted drug development is essential, but at the moment, the academic computer models are constrained by imprecise datasets or a lack of knowledge about the underlying molecular mechanisms of the disease they are meant to cure. Further experimentation must be done via in-vitro studies to proceed with the drug development process, followed by clinical trials to assess safety, dosage and efficacy in humans, which is then reviewed and ready for post market safety monitoring.

## Conclusion:

The selected thiazo derivatives are showing better docking interaction (-8.4 to - 6.6 kcal) compared to clinically proven drugs (-6.7 and - 6.1 kcal). All the compounds are obeying Lipinski's rule of five and similar toxicity profiles like sulfamethoxazole. These compounds are proven to be potential inhibitors for the cystalysin protein of *Treponema denticola*.

## Figures and Tables

**Table 1:** Molecular docking scores and residual amino acid interactions of Imidazole Quinoline compounds (1-6) against virulence factor gingipain R (RgpB) of *Porphyromonas gingivalis* (PDB ID – 1CVR)

Ligands	Docking scores/Affinity (kcal/mol)	H-bond	Amino Acid Residual interactions	
			Hydrophobic/Pi-Cation	Van dar Waals
1	-7.4	Tyr-123, Ser-237, Ala-243	Val-99, Val-98, Lys-238, Ile-205, Phe-103, Pro-100	Arg-107, Asp-203, Gly-97, Ala-96, Met128
2	-8.4	Tyr-124, Arg-107	Phe-103, Pro-125, Val-99, Val-38, Tyr-123, Leu-17	Ala-39
3	-7.8	Tyr-123, Ser-237	Phe-103, Ile-205, Val-99, Pro-100, Gly-97	Arg-107, Lys-238, Gly-246, Met-247
4	-7.2	Ser-237	Pro-100, Ala-243, Val-99, Phe-103	Arg-107, Lys-238, Met-128
5	-8.2	Tyr-124	Pro-100, Phe-103, Pro-125, Val-99, Val-38, Leu-17	Ala-39, Tyr-123, Arg-107
6	-6.6	Ala-39, Lys-238, Ala-96	Val-99, Ala-243, Tyr-124, Asp-355	Val-38, Pro-100, Gly-97, Ile-205, Arg-369
Azithromycin	-6.7	Tyr-124, Tyr-123	Ile-205, Val-98, Val-99	Leu-17, Leu-21, Val-38, Ala-39, Ala-243, Ser-237, Gly-97, Met-247
Sulfanilamide	-4.9	Arg-107, Pro-100	Phe-103	Asn-104, Ser-269, Thr-268
Sulfamethoxazole	-6.1	Met-247, Ser-248, Tyr-123	Ala-39, Val-38, Val-98, Ser-237, Ala-243	Ala-235, Gly-97, Val-99, Tyr-124, Arg-369

**Table 2:** Swiss ADME values of selected Imidazole Quinoline compounds (1-6)

Compound	log Kp (cm/s)	GI absorption	BBB permeant	Pgp substrate	CYP1A2 inhibitor	CYP2C19 inhibitor	CYP2C9 inhibitor	CYP2D6 inhibitor	CYP3A4 inhibitor
1	-5.97	Low	No	Yes	No	Yes	Yes	No	Yes
2	-4.96	Low	No	No	No	Yes	Yes	No	No
3	-6.32	Low	No	Yes	No	No	Yes	No	Yes
4	-5.55	Low	No	No	No	Yes	Yes	No	No
5	-5.18	Low	No	No	No	Yes	Yes	Yes	No
6	-8.04	Low	No	Yes	No	No	No	No	No
Azithromycin	-8.01	Low	No	Yes	No	No	No	No	No
Sulfanilamide	-7.79	High	No	No	No	No	No	No	No
Sulfamethoxazole	-7.21	High	No	No	No	No	No	No	No

**Table 3:** Lipinski and Veber rules of selected Imidazole Quinoline compounds (1-6)

Compound	MW	iLogP	HBD (nOHNH)	HBA (nON)	nrotb	MR	TPSA	Lipinski #violations	Bio availability score
Lipinski*	≤500	≤5	≤5	≤10	≤10	-	-		
Veber**	-	-	-	-	-	-	≤ 140		
1	478.56	0	2	7	8	130	116.57	0	0.56
2	417.48	3.1	2	5	5	118.67	99.5	0	0.55
3	452.53	2.97	2	7	6	121.1	140.36	0	0.56
4	367.42	2.38	2	5	5	101.16	99.5	0	0.55
5	416.5	2.92	2	4	5	121.05	105.29	0	0.55
6	432.45	2.33	3	9	7	117.5	168.7	1	0.55
Azithromycin	748.98	4.76	5	14	7	200.78	180.08	2	0.17
Sulfanilamide	172.2	0.61	2	3	1	41.84	94.56	0	0.55
Sulfamethoxazole	253.28	1.03	2	4	3	62.99	106.6	0	0.55

**Table 4:** Toxicity profile of selected Imidazole Quinoline compounds (1-6)

Compound	^a^LD_50_ (mg/kg)	Class	Toxicity				
			HEPATOTOXICITY	CARCINOGENICITY	IMMUNOTOXICITY	MUTAGENICITY	CYTOTOXICITY
1	2000	4	Inactive	Inactive	Inactive	Inactive	Inactive
2	5000	5	Inactive	Active	Inactive	Inactive	Inactive
3	1500	4	Active	Active	Inactive	Inactive	Inactive
4	5000	5	Inactive	Active	Inactive	Inactive	Inactive
5	5000	5	Inactive	Active	Inactive	Inactive	Inactive
6	12500	6	Inactive	Active	Inactive	Inactive	Inactive
Azithromycin	2000	4	Inactive	Inactive	Active	Inactive	Inactive
Sulfanilamide	3000	5	Inactive	Active	Inactive	Inactive	Inactive
Sulfamethoxazole	2300	5	Active	Active	Inactive	Inactive	Inactive
